# Safety-Oriented Evaluation of Large Language Models in Health Care: Guideline-Informed Systematic Review

**DOI:** 10.2196/97240

**Published:** 2026-08-20

**Authors:** Hikaru Matsuoka, Takayuki Takahashi, Takayuki Semitsu, Keiko Matsuoka, Ryoma Hayashi

**Affiliations:** 1 Department of Information Security, Faculty of Information Systems, University of Nagasaki, Nagasaki, Nagasaki, Japan; 2 Information-Technology Promotion Agency, Minato, Tokyo, Japan; 3 Statistical Genetics Team, RIKEN Center for Advanced Intelligence Project, Nihonbashi 1-chome Mitsui Building 15F, 1-4-1 Nihonbashi, Chuo-kuTokyo, 103-0027, Japan, 81 3-6225-2482; 4 Department of Obstetrics and Gynecology, Keio University School of Medicine, 35 Shinanomachi, Shinjuku-ku, Tokyo, Japan; 5 Department of Obstetrics and Gynecology, Shonan Atsugi Hospital, Atsugi, Kanagawa, Japan

**Keywords:** artificial intelligence, AI, large language model, patient safety, systematic review, trustworthy artificial intelligence, trustworthy AI, health care informatics, clinical decision support, guideline, evaluation framework, public health informatics

## Abstract

**Background:**

Large language models (LLMs) are rapidly emerging in health care, offering opportunities in decision support, education, and research, but raising critical concerns about safety, reliability, and ethics. Although several guidelines for trustworthy AI exist in business and technology, few systematic reviews have applied them to medical contexts.

**Objective:**

This study aimed to conduct a systematic review of LLM research in health care, applying the AI Guidelines for Business as a framework across 11 domains, including safety, reliability, ethics, transparency, fairness, inclusiveness, privacy, security, robustness, data quality, and verifiability.

**Methods:**

Following PRISMA (Preferred Reporting Items for Systematic Reviews and Meta-Analyses) 2020 guidelines (retrospectively registered on the Open Science Framework; DOI 10.17605/OSF.IO/P4KSB), the PubMed, Scopus, Web of Science, arXiv, and IEEE Xplore databases were searched on January 15, 2025. Records were screened in 2 stages by 3 reviewers (with records retained only upon unanimous agreement). A total of 247 studies were included, of which 211 (85.4%) contributed quantitative values. Eligible studies were classified across 11 trustworthy AI domains. Heterogeneous metrics were summarized within metric families; when multiple models were evaluated, the mean across models was used as the primary estimate, with best, median, and primary-model sensitivity analyses. The LLM-assisted categorization (GPT-5 mini) was validated by using an automated internal consistency check, and 95% CIs were estimated by using cluster bootstrap on study-level values.

**Results:**

Of the 25,156 records, 247 (1.0%) studies were included, and of these, 211 (85.4%) contributed quantitative values. Evaluation concentrated on accuracy (143/247, 57.9%) and fairness and inclusiveness (93/247, 37.7%), followed by data quality (47/247, 19.0%) and prevention of misinformation (44/247, 17.8%). Normalized performance was moderate to high (accuracy mean 0.73, 95% CI 0.7-0.76; data quality: 0.64; prevention of misinformation: 0.82). Selecting the best-performing model inflated domain means by up to 0.05. Privacy protection (2/247, 0.8%) and security assurance (0/247, 0.0%) were almost entirely absent. Domain assignments were recoverable from objective metric types in 98.9% of values (Cohen κ=0.985).

**Conclusions:**

Current evaluations emphasize accuracy while underreporting privacy, security, robustness, explainability, and verifiability. This finding reflects gaps in reporting rather than demonstrated poor performance, underscoring the need for comprehensive, guideline-based, multidomain evaluation before deployment in high-stakes clinical settings.

## Introduction

The adoption of large language models (LLMs) within health care has accelerated sharply, fueled by advances in generative AI and the growing demand for tools to support diagnostics, clinical documentation, patient communication, medical education, and clinical decision-making [1-4]. The potential of these models to synthesize vast amounts of medical knowledge and streamline clinical workflows is significant [5,6]. Yet, alongside this promise, concerns over their safety, reliability, ethical implications, fairness, transparency, robustness, accountability, privacy, and overall trustworthiness have become increasingly salient [7-10]. Although many early studies focused on assessing the diagnostic accuracy or utility of LLMs, particularly on standardized examinations [11,12], fewer have systematically evaluated whether these models conform to comprehensive guideline-based standards for responsible AI or whether domains such as safety and ethical risk are adequately addressed in practice [13,14].

Recent systematic reviews have begun to shed light on these multifaceted issues. For instance, assessments of LLM performance under examination conditions reveal moderate to high accuracy but also significant variability across studies and medical specialties [15,16]. Other research highlights the critical risks to patient safety and the increased clinician burden that can arise from the uncritical deployment of generative AI, emphasizing the need for robust harm reduction strategies [17,18]. Studies exploring user perceptions in specialized fields such as mental health underscore the importance of safety guardrails and technical controls to mitigate potential harms [19]. Furthermore, critical assessments of the use of LLMs in research, such as their use in systematic reviews, have identified substantial methodological challenges and a high risk of factual errors or hallucinations, which could compromise the integrity of evidence synthesis [20,21]. Together, these studies demonstrate that although performance benchmarks are steadily advancing, structured and comprehensive safety assessment remains a critical but underdeveloped area [13].

Concurrently, a robust ecosystem of guideline frameworks has emerged from academic, industry, and international initiatives to define the principles of trustworthy and responsible AI. Reporting guidelines such as CONSORT (Consolidated Standards of Reporting Trials)-AI and SPIRIT (Standard Protocol Items: Recommendations for Interventional Trials)-AI provide frameworks for standardizing the evaluation of clinical trials involving AI interventions, promoting transparency and reproducibility [22,23]. The FUTURE (Fairness, Universality, Traceability, Usability, Robustness, and Explainability)-AI guideline offers an international consensus on 6 core principles—fairness, universality, traceability, usability, robustness, and explainability, as well as best practices for the entire lifecycle of medical AI systems, particularly in medical imaging [24]. Broader mapping studies, including a review of 200 guidelines and recommendations for AI governance, have identified recurring ethical principles across international frameworks [25]. In Japan, the AI Guidelines for Business, jointly compiled by the Ministry of Internal Affairs and Communications and the Ministry of Economy, Trade and Industry, similarly highlight key areas for responsible implementation, including safety, reliability, ethics, transparency, and data quality [26].

This development of best practices is mirrored by growing regulatory momentum worldwide. In the European Union, the AI Act is set to impose stringent, risk-based requirements on high-risk AI systems—a category that includes many health care applications—mandating rigorous risk assessment, transparency, conformity, and postmarket monitoring [27]. In the United States, the Food and Drug Administration (FDA) is actively advancing its regulatory framework for AI- or machine learning (ML)–based medical devices, emphasizing the need for a total product lifecycle approach to ensure safety and effectiveness [28]. At the global level, the World Health Organization (WHO) has published landmark guidance emphasizing that AI systems for health must be designed to respect human rights, mitigate bias, ensure safety and privacy, and maintain clear lines of accountability [29]. These convergent developments reflect a clear global expectation that medical LLMs must not only perform technical tasks effectively but also adhere to a stringent set of ethical, safety, regulatory, and trustworthiness criteria.

We adopted the AI Guidelines for Business as the organizing framework for 3 reasons. First, unlike most health-specific reporting standards, which target specific study types (eg, CONSORT-AI and SPIRIT-AI for trials, TRIPOD (Transparent Reporting of a Multivariable Prediction Model for Individual Prognosis or Diagnosis)+AI for prediction models, and CLAIM (Checklist for Artificial Intelligence in Medical Imaging) and STARD (Standards for Reporting Diagnostic Accuracy Studies)-AI for diagnostic and imaging studies), the framework provides a single, cross-sectoral taxonomy spanning the full range of trustworthy AI principles, allowing heterogeneous LLM evaluation studies to be mapped onto 1 consistent scheme. Second, its principle set aligns closely with international health guidance, including WHO guidance and FUTURE-AI; we make this alignment explicit in a correspondence table (Multimedia Appendix 1). Third, it is implementation and lifecycle oriented, making it well suited to a review concerned with deployment readiness. We use it as a complementary organizing scaffold rather than a substitute for health-specific reporting guidelines, against which we cross-map every domain.

This study aims to fill these gaps by conducting a systematic review of the medical LLM literature, using a comprehensive, business-derived AI guidelines framework. We will classify existing studies across multiple domains, including safety, reliability, ethics, transparency, fairness, privacy, accountability, explainability, robustness, data quality, inclusiveness, and verifiability, using structured prompts for data extraction. Furthermore, we will produce descriptive visualizations to illustrate how frequently each domain is addressed, thereby highlighting areas of considerable risk or deficiency and delineating critical research gaps. The findings aim to inform safer, more trustworthy medical LLM development, regulation, and clinical deployment. For contextual comparison, we incorporate the review methodology of Shool et al [16], focusing on the extraction and assessment of numerical data to ensure that our synthesis remains focused on objective evidence while aligning with prior systematic efforts, thereby transparently capturing the limitations of current evaluation metrics.

## Methods

### Systematic Review Data Extraction and Management Protocol

The literature was systematically searched on January 15, 2025, using the PubMed, Scopus, Web of Science, arXiv, and IEEE Xplore databases. Queries combined relevant keywords and MeSH terminology such as evaluation, LLMs, AI chatbot, and medical and clinical practice. The complete search schema is presented in Multimedia Appendix 2; the title-field searches yielded 1201 (4.8%) records in PubMed, 3273 (13.0%) records in Scopus, 1989 (7.9%) records in Web of Science, 18,128 (72.1%) records in arXiv, and 565 (2.2%) records in IEEE Xplore (N=25,156 records). This review followed PRISMA (Preferred Reporting Items for Systematic Reviews and Meta-Analyses) 2020 (completed checklist in Checklist 1) and was retrospectively registered on the Open Science Framework (OSF; DOI 10.17605/OSF.IO/P4KSB; registered on June 22, 2026; labeled retrospective), as it was not prospectively registered. Eligible studies were original research that empirically evaluated ≥1 LLM in a health care context and reported at least 1 quantitative outcome amenable to scoring, published in English; nonoriginal work, records with inaccessible text or a missing DOI, duplicate records, and studies without original quantitative evaluation were excluded. Preprints were identified by their archive URL (arXiv or medRxiv) and examined in a sensitivity analysis. Records were screened in 2 stages (title and abstract, followed by full-text screening) by 3 reviewers working together; records were retained only when all 3 reviewers agreed (ie, by unanimous consensus).

A comprehensive search across the PubMed, Scopus, Web of Science, arXiv, and IEEE Xplore databases yielded 25,156 records, from which 2754 (10.9%) duplicate records and 328 (1.3%) additional records were removed. This resulted in 22,074 (87.7%) records being screened by title and abstract, leading to the exclusion of 20,198 (91.5%) records that did not meet the inclusion criteria. Following this, data extraction was performed on 1876 (8.5%) articles that passed the initial screening. Of these, 586 (31.2%) articles were excluded because of inaccessible abstracts or full texts, missing DOIs, duplication, and nonoriginal research designs.

Following a detailed full-text review, an additional 529 (41.0%) articles were excluded. Ultimately, this rigorous and meticulous effort culminated in 761 (59.0%) articles from which data were fully extracted. After further excluding studies without original quantitative evaluation or for which reliable scoring was not feasible, a final dataset of 247 (32.5%) articles was retained for analysis.

### Approach to Score Calculation and Normalization

We summarized performance within metric families (percentages, 0-1 proportions, Likert or ordinal, readability, and agreement) and did not pool fundamentally different families; metric-family-specific summaries are provided in Multimedia Appendix 3. For an auxiliary descriptive comparison only, extracted values were normalized to a 0-1 scale. Performance measures such as accuracy, the area under the curve (AUC), and the *F*_1_-score were regarded as equivalent indicators of correctness and were normalized accordingly. For Likert-type scales (eg, 5-point scales), scores were rescaled such that the highest value (eg, 5) corresponded to the most favorable outcome. For readability indices, including the Simple Measure of Gobbledygook (SMOG) index and the Flesch-Kincaid Grade Level, the maximum level of 21, representing graduate-level complexity, was provisionally defined as the upper bound, and scores were aggregated relative to this reference point.

When a study evaluated multiple models, we applied a prespecified extraction rule and computed a per-study representative value as the mean across models (the primary estimate), with the best, median, and primary (first-reported) model used as sensitivity analyses (Multimedia Appendix 3). The analysis was descriptive, not a formal meta-analysis; to avoid pseudoreplication, we aggregated data into 1 value per study per domain and estimated 95% CIs by using cluster bootstrap resampling of studies (with categories containing <5 valid items excluded from interval estimation). For the branch-of-medicine subgroup analyses, which are based on the best-model value rather than the mean across models, 95% CIs were calculated as mean ± 1.96 × SD/√n using a normal approximation; these intervals may therefore extend beyond the 0-1 range of the normalized score.

### Data Classification and Categorization

The number of publications increased between 2019 and 2025. Of the 761 articles from which data were fully extracted, 1 (0.1%) was published in 2019, followed by 3 (0.4%) in 2020, 6 (0.8%) in 2021, 7 (0.9%) in 2022, and 160 (21.0%) in 2023. The largest number was published in 2024, with 557 (73.2%) articles, followed by 27 (3.5%) in early 2025. These are descriptive publication-year counts of the 761 articles; no statistical test for change over time was performed.

In the process of study selection and data extraction, each article was analyzed to determine the corresponding medical specialty (allowing for multiple assignments), 3 independent binary indicators (emergency conditions, surgical diagnosis, and medical [internal medicine] diagnosis), which are not mutually exclusive (ie, a study may be flagged for >1), as well as the intended purpose of use and the study population. An LLM (GPT-5 mini [gpt-5-mini, OpenAI]) was used as an auxiliary tool for text categorization. We note the timeline explicitly: the literature search was conducted on January 15, 2025, whereas LLM-assisted extraction and categorization were performed in September 2025, after the public release of the GPT-5 model family on August 7, 2025; the model (GPT-5 mini), access date (September 2025), default decoding temperature, and full prompts are reported in Multimedia Appendix 4. Terms identified in each study were mapped to the categories defined in the AI Guidelines for Business and the Guide to Evaluation Perspectives on AI Safety by using the coding manual in Multimedia Appendix 5. To validate the categorization without additional human raters, we used an automated internal consistency check: domain assignments matched the metric-derived domain in 98.9% of 3646 extracted values (Cohen κ=0.985; 99.0% of 1454 distinct metric types mapped to a single domain), indicating that the categorization was systematic and reproducible. Because conventional risk-of-bias tools (eg, PROBAST [Prediction Model Risk of Bias Assessment Tool]-AI) require subjective expert judgment that does not scale across this large number of cross-specialty studies, we instead derived an objective Reporting Transparency Index (RTI) from the extracted data (model named, version or date specified, sample size reported, ≥2 models compared, and ≥2 distinct metrics reported; score range 0-5) and used it in a sensitivity analysis restricted to higher-transparency studies (Multimedia Appendix 6).

## Results

### Study Selection

**Figure 1.**
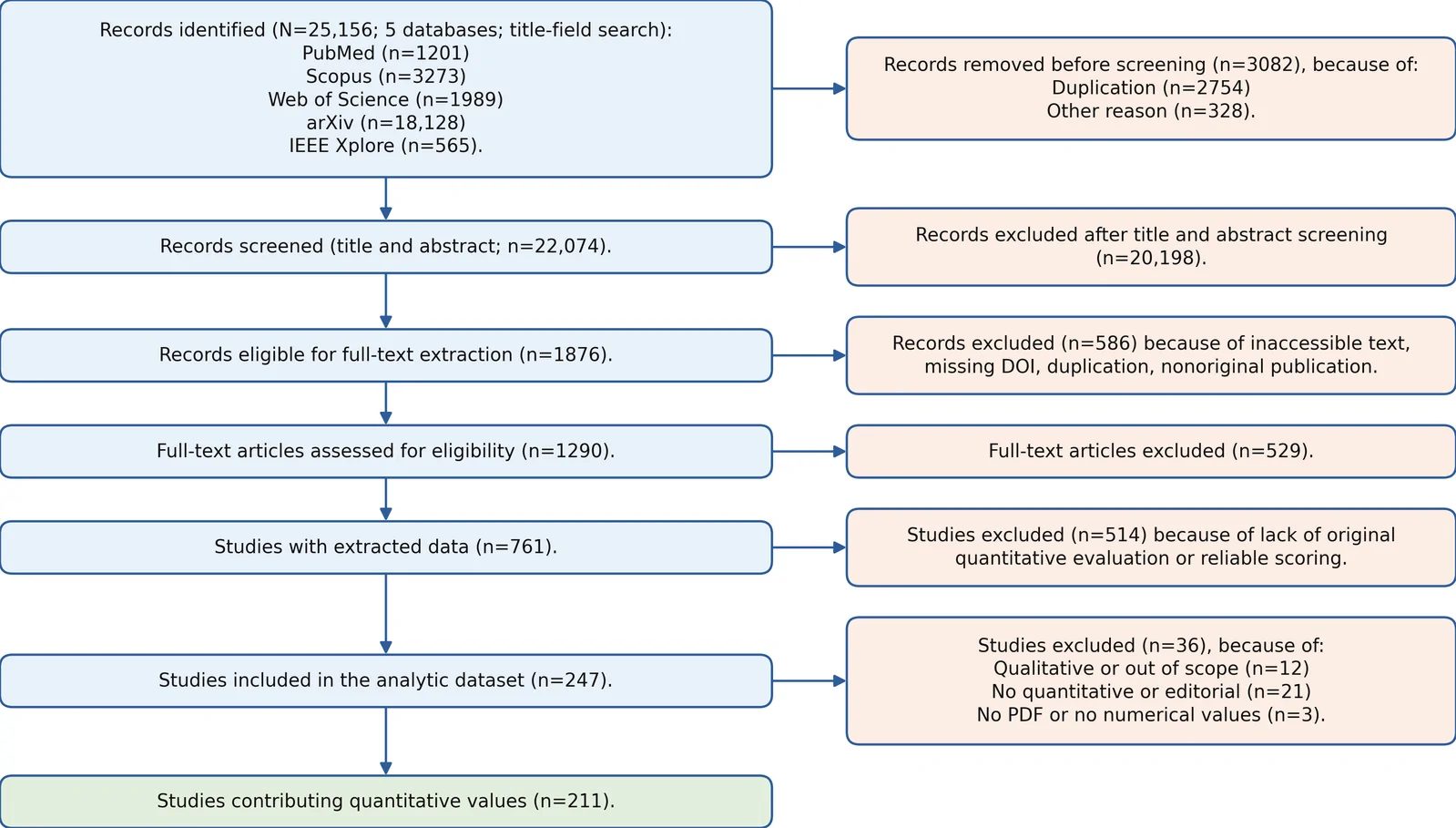
PRISMA (Preferred Reporting Items for Systematic Reviews and Meta-Analyses) 2020 flow diagram showing study identification, screening, and inclusion. Of 25,156 records across 5 databases (title-field searches), 247 studies were included, of which 211 contributed extractable quantitative values.

The study selection process is summarized in Figure 1. Of the 25,156 records identified across the 5 databases, 247 studies met the inclusion criteria, of which 211 contributed extractable quantitative values.

### Distribution of Categories

**Figure 2.**
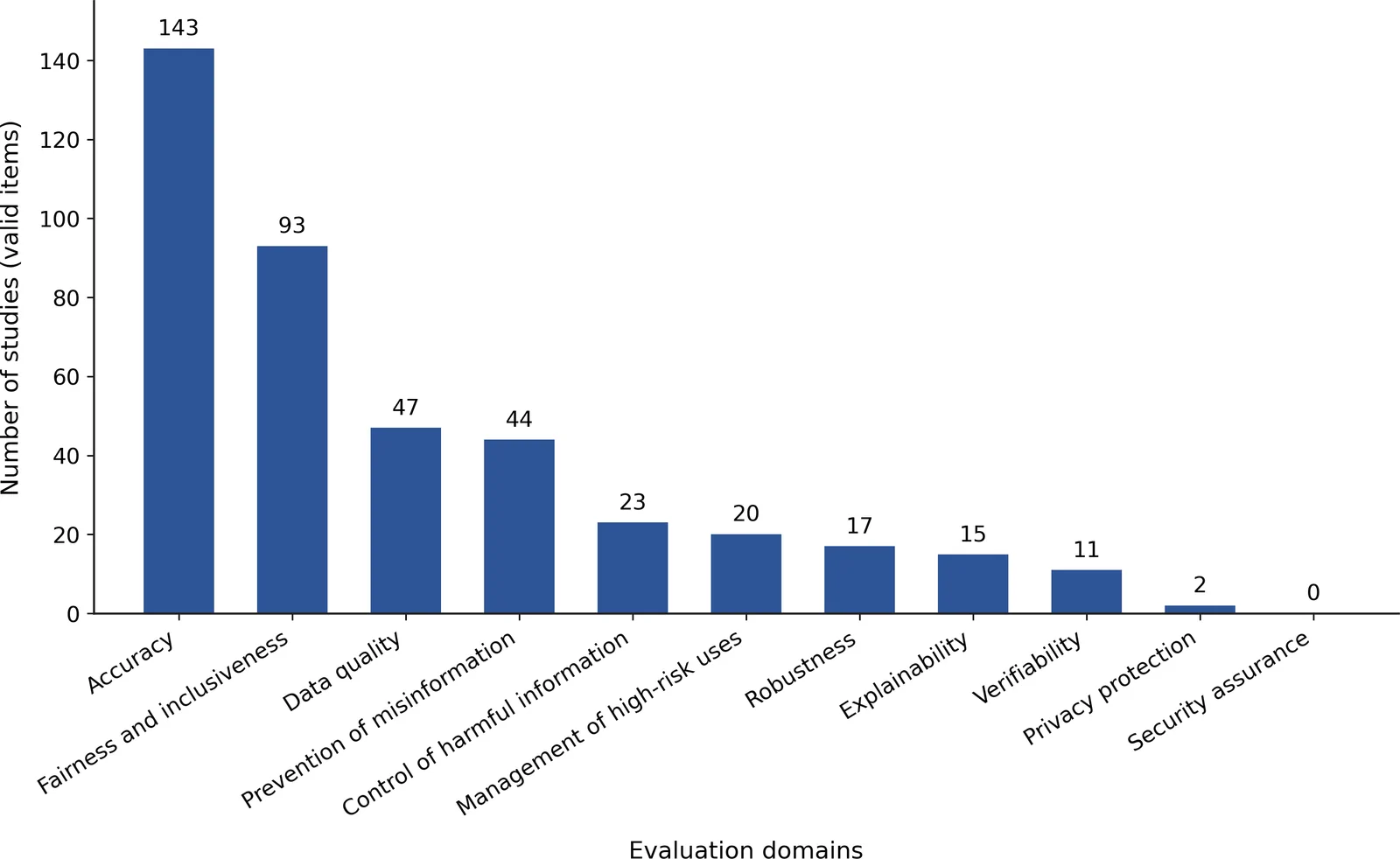
Number of studies addressing each trustworthy AI evaluation domain (N=247). Accuracy was the most frequently assessed domain; privacy protection and security assurance domains were almost absent.

Figure 2 presents the number of papers across multiple categories. A valid item denotes a study-domain pair with an extractable quantitative value; of the 247 included studies, 211 (85.4%) contributed valid items. This highlights which categories were more frequently addressed and which were largely overlooked.

On the basis of the terminology exemplified in Table 1, the reported scores for each category were extracted as assessed by the authors. Quantitative metrics such as accuracy, AUC, and the *F*_1_-score provide objective indicators of model performance and allow consistent comparison across studies. Their use facilitates transparency and reliability in evaluating LLMs for health care applications.

**Table 1.** Category-keyword correspondence used to map extracted terms to trustworthy AI domains (full coding manual in Multimedia Appendix 5).

Trustworthy AI domains	Representative terms or keywords used for mapping
Safety (overall)	safety
Control of harmful information	harm
Prevention of misinformation	misinformation; misuse; usefulness; relevance; reliability
Fairness and inclusiveness	bias; fair; acceptability; concise; comprehensive
Management of high-risk uses	risk
Privacy protection	privacy
Security assurance	security
Explainability	readability; transparency; visibility; understandability; clarity
Robustness	robust; consistency; coherence; redundancy
Data quality	quality; completeness
Verifiability	verification; actionability; reference

Accuracy is the most represented category, with 143 valid items, showing that performance measurement is the most commonly reported aspect. Fairness and inclusiveness (93 valid items), data quality (47 valid items), and prevention of misinformation (44 valid items) follow as moderately covered categories. Categories such as control of harmful information (23 valid items), management of high-risk uses (20 valid items), robustness (17 valid items), explainability (15 valid items), and verifiability (11 valid items) have relatively limited coverage, indicating less consistent reporting. Privacy protection (2 valid items) and security assurance (0 valid items) are almost entirely absent, suggesting that these categories have received comparatively little attention within the field of health care.

### Category-Wise Numerical Evaluation

**Figure 3.**
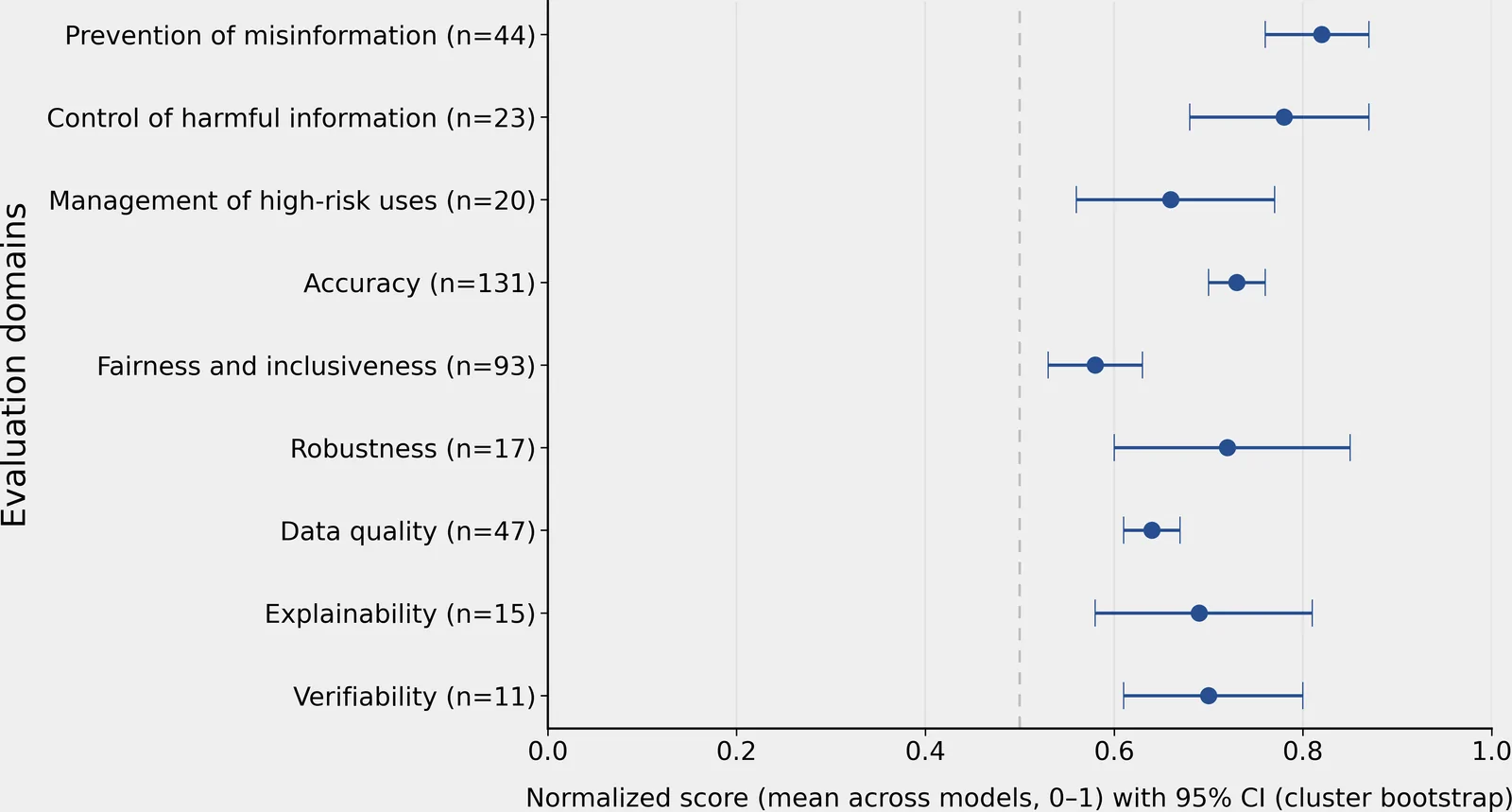
Domain-level performance showing study-aggregated mean normalized score (0‐1; mean across models) with 95% CIs estimated by cluster bootstrap. Domains with <5 valid items (privacy and security) are not shown.

A descriptive interval plot (Figure 3) presents study-aggregated mean scores and 95% CIs (cluster bootstrap) for each evaluation category. Categories with <5 valid items were excluded to avoid unstable estimates. Reporting metric families separately revealed substantial within-domain heterogeneity (eg, the normalized median for accuracy differed by family: Likert or ordinal, 0.85; percentage, 0.72; 0-1 proportion, 0.76; Multimedia Appendix 3).

Accuracy (131/247, 53.0%; mean 0.73, 95% CI 0.7-0.76) had the largest sample size and a narrow CI. Selecting the best-performing model rather than averaging inflated domain means by 0.01 to 0.05 (eg, accuracy best 0.78 vs mean 0.73); therefore, we report mean-based estimates as the primary analysis (Multimedia Appendix 3). Restricting the synthesis to higher-transparency studies (RTI≥3; 186/242) changed domain estimates by at most 0.004, and excluding the 17 preprints in the analytic set changed estimates by at most 0.018, indicating robustness. Control of harmful information (23/247, 9.3%), prevention of misinformation (44/247, 17.8%), and data quality (47/247, 19.0%) demonstrated relatively high mean scores (0.64-0.82) with moderate precision. Fairness and inclusiveness (93/247, 37.7%; mean 0.58, 95% CI 0.53-0.63) showed a lower average score but maintained a reasonably narrow CI because of the larger sample size.

Management of high-risk uses (20/247, 8.1%; mean 0.66, 95% CI 0.56-0.77), explainability (15/247, 6.1%; mean 0.69, 95% CI 0.58-0.81), robustness (17/247, 6.9%; mean 0.72, 95% CI 0.6-0.85), and verifiability (11/247, 4.5%; mean 0.7, 95% CI 0.61-0.8) all exhibited wide CIs. The wide intervals reflect considerable variability and reduced certainty of the estimates. This is mainly attributable to the small number of valid items in these categories, which made the results more sensitive to outliers and sampling error.

In summary, categories supported by larger sample sizes (such as accuracy) yielded stable estimates with narrow CIs, while categories with limited representation (explainability, robustness, verifiability, and management of high-risk uses) produced much wider CIs, highlighting substantial variability and uncertainty.

### Evaluations Within Each Branch of Medicine

#### Internal Medicine

**Figure 4.**
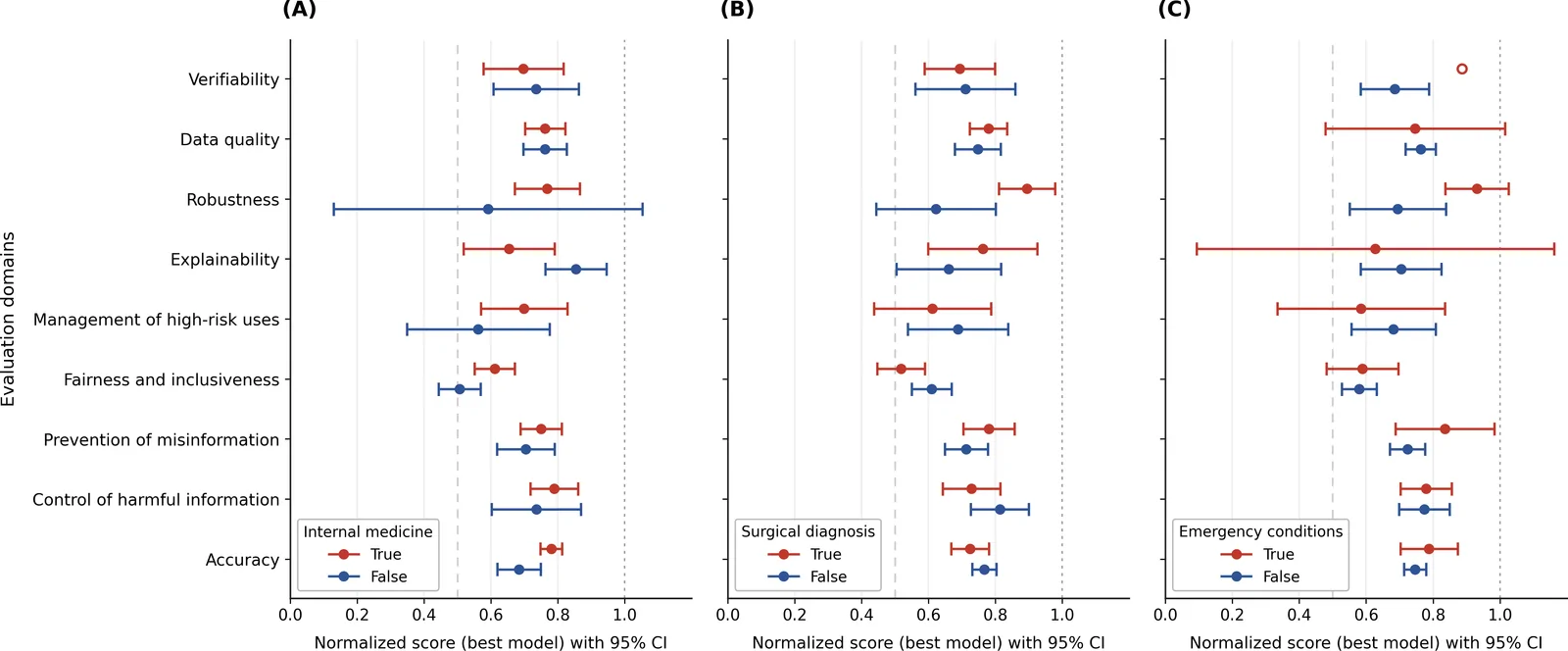
Performance by branch-of-medicine subgroup (true vs false) for (A) internal medicine, (B) surgical diagnosis, and (C) emergency conditions. Points represent subgroup means (best-model value) with 95% CIs.

Here, the True and False subgroup labels denote whether each independent binary indicator (emergency, surgical, or medical diagnosis) applied to a study. In the internal medicine group (Figure 4A), accuracy was consistently high, particularly for the True subgroup (98/140, 70.0%; mean 0.78, 95% CI 0.75-0.81), although slightly lower for the False subgroup (42/140, 30.0%; mean 0.68). Control of harmful information and prevention of misinformation also showed moderate to high scores (means ~0.75), but the estimates were based on relatively smaller sample sizes and therefore displayed wider CIs. Notably, fairness and inclusiveness achieved relatively higher scores in this domain, especially in the True subgroup (66/93, 71.0%; mean 0.61, 95% CI 0.55-0.67), which stands out compared with other categories and indicates a greater emphasis on equitable evaluation within internal medicine. Data quality was another strong category, with both True and False subgroups showing mean values of approximately 0.76 and narrow CIs; small-sample categories are interpreted with caution and not described as robust. Categories such as robustness (mean 0.77) and verifiability (mean 0.7) were also represented, although the smaller number of cases led to broader variability. By contrast, management of high-risk uses and explainability were less well supported, with only limited data and correspondingly wide CIs.

#### Surgical Diagnosis

In the surgical diagnosis group, accuracy remained high in both subgroups (True: 50/140, 35.7%; mean 0.72 and False: 90/140, 64.3%; mean 0.77), with relatively narrow CIs. Control of harmful information and prevention of misinformation also yielded favorable means of around 0.73 to 0.81, although they were based on modest sample sizes. Fairness and inclusiveness showed moderate values, with the False subgroup (64/93, 68.8%; mean 0.61, 95% CI 0.55-0.67) slightly higher than the True subgroup (29/93, 31.2%; mean 0.52). Data quality was consistently strong across both groups (means ~0.75-0.78). Robustness presented mixed results: although the True subgroup reached a notably high mean of 0.89, this estimate was derived from only 6 cases and thus carried wide uncertainty, whereas the False subgroup mean was lower (0.62). Other categories such as management of high-risk uses, explainability, and verifiability were represented by <15 cases and exhibited wider intervals, reflecting reduced reliability.

Overall, the interval plot (Figure 4B) demonstrates that surgical diagnosis evaluations emphasize accuracy and data quality, with moderate attention to fairness. However, caution is warranted when interpreting categories with very limited sample sizes, for which variability remains substantial.

#### Emergency Conditions

In the emergency conditions group, accuracy remained high across both subgroups, with the True group (17/140, 12.1%; mean 0.79; 95% CI 0.7-0.87) showing slightly higher values than the False group (123/140, 87.9%; mean 0.75; 95% CI 0.71-0.78). Control of harmful information (18/22, 81.8%; mean 0.77) and prevention of misinformation (38/43, 88.4%; mean 0.72) demonstrated moderate to strong performance, although sample sizes were relatively limited for the former. Fairness and inclusiveness showed balanced values across groups (True: 0.59, False: 0.58), suggesting consistent but moderate attention to equity. Data quality (43/46, 93.5%; mean 0.76, 95% CI 0.72-0.81) again emerged as a reliable category, with narrower CIs than those of other measures. By contrast, categories such as management of high-risk uses, explainability, robustness, and verifiability were represented by <15 cases, leading to wider intervals and greater uncertainty regarding their interpretation.

Overall, the interval plot (Figure 4C) indicates that emergency condition evaluations prioritize accuracy and data quality, while fairness remains moderate. Importantly, the evaluation of LLM performance in emergency contexts is particularly meaningful, as these scenarios often involve time-critical decisions for which reliability and safety are paramount.

### Application and Study Population

**Figure 5.**
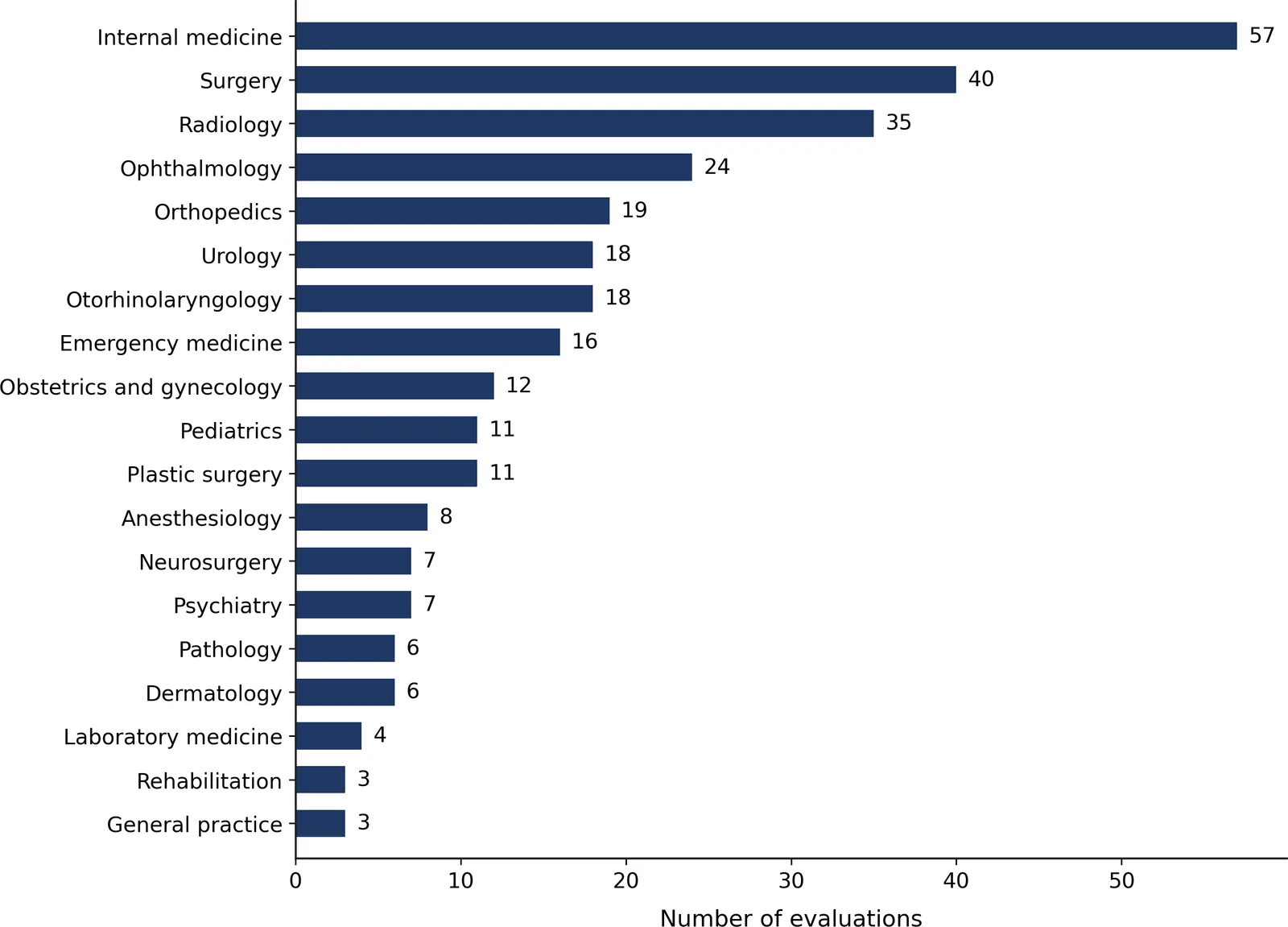
Distribution of evaluations across 19 predefined medical specialties.

The 19 predefined medical specialties were categorized using an LLM according to the structured prompt outlined in Table 2. The distribution of evaluations across the 19 medical specialties (Figure 5) revealed substantial variation in research focus. Internal medicine accounted for the largest share (57/247, 23.1%), highlighting its central role in the assessment of LLMs. This was followed by surgery (40/247, 16.2%) and radiology (35/247, 14.2%), indicating strong representation in procedure-oriented and imaging-based fields. Ophthalmology (24/247, 9.7%), orthopedics (19/247, 7.7%), and urology (18/247, 7.3%) also contributed considerably, alongside otorhinolaryngology (18/247, 7.3%) and emergency medicine (16/247, 6.5%).

**Table 2.** Structured data extraction schema applied with a large language model (LLM)–assisted prompt (full prompt in Multimedia Appendix 4).

Extraction fields	Description or allowed values
Study population	Free text (eg, adult patients with type 2 diabetes; emergency physicians)
Medical specialty (multiple allowed)	≥1 of the following 19 specialties: internal medicine; pediatrics; dermatology; psychiatry; surgery; orthopedics; obstetrics and gynecology; ophthalmology; otorhinolaryngology; urology; neurosurgery; radiology; anesthesiology; pathology; laboratory medicine; emergency medicine; plastic surgery; rehabilitation; general practice
Emergency conditions	True or false (independent indicator)
Surgical diagnosis	True or false (independent indicator)
Medical (internal medicine) diagnosis	True or false (independent indicator)
Application	Free text (intended use; eg, clinical decision support)
LLMs evaluated	Model names and version
Reported metrics and values	For example, accuracy, area under the curve, *F*_1_-score, Likert rating, readability index
Trustworthy AI domains	Mapped according to Table 1 (multiple domains allowed)

Midrange representation was observed for obstetrics and gynecology (12/247, 4.9%), pediatrics (11/247, 4.5%), and plastic surgery (11/247, 4.5%), reflecting moderate engagement. Several specialties showed lower counts, including anesthesiology (8/247, 3.2%), neurosurgery (7/247, 2.8%), and psychiatry (7/247, 2.8%). At the lowest end, pathology (6/247, 2.4%), dermatology (6/247, 2.4%), laboratory medicine (4/247, 1.6%), rehabilitation (3/247, 1.2%), and general practice (3/247, 1.2%) were minimally represented.

Overall, the graph demonstrates that LLM-related evaluations have concentrated heavily in internal medicine, surgery, and radiology, while primary care and supportive specialties remain underexplored. This imbalance underscores the need for broader coverage to ensure that AI applications are evaluated across the full spectrum of medical practice.

## Discussion

### Principal Results

This systematic review reveals a marked imbalance in the evaluation of medical LLMs. Our findings demonstrate that the vast majority of current research is hyper-focused on performance metrics, with accuracy being the most dominant and consistently reported domain (143/247, 57.9%; mean 0.73). Although demonstrating technical capability is a necessary first step, this narrow focus comes at the expense of the foundational principles required for trustworthy AI. Critically, core domains such as safety, privacy, and security were almost entirely absent from the literature reviewed. This evaluation gap signifies a notable gap between the rapid growth in publications and the comprehensive, multidomain assessment needed before these technologies can be responsibly integrated into clinical practice. Importantly, the near-absence of privacy and security reflects a lack of evaluation and reporting rather than demonstrated poor performance; this review characterizes reporting patterns and does not audit deployed systems or clinical outcomes.

The issue of fairness and inclusiveness presents a noteworthy tension. Although it was the second most frequently assessed nonaccuracy domain (93/247, 37.7%), it consistently yielded the lowest average performance score (mean 0.58). This suggests that although the research community is beginning to acknowledge the importance of mitigating bias, current models still struggle considerably to generate equitable and inclusive outputs. Our analysis further revealed that this problem was especially pronounced in the surgical domain. Studies evaluating LLMs for surgical diagnoses reported a substantially lower mean fairness score (0.52) than those in internal medicine (0.61). This disparity may reflect underlying biases in surgical training data, demographic imbalances in procedural literature, or the nuanced language required for surgical decision-making, highlighting a critical area for targeted improvement.

The limited reporting and wide CIs for other essential categories, such as robustness, explainability, and verifiability, further underscore the immaturity of current evaluation methodologies. The high variability in these results indicates a lack of standardized metrics and a superficial approach to assessing model reliability.

### Limitations

This study has several limitations that warrant consideration. Primarily, the LLM-assisted categorization was validated by a fully automated internal consistency check rather than against an independent human gold standard, so residual misclassification, particularly for low-prevalence domains, cannot be fully excluded. In place of a subjective risk-of-bias assessment, we used an objective RTI, which captures completeness of reporting rather than internal validity per se. This review was not prospectively registered (it was retrospectively registered on OSF), and screening was not independent or duplicated. Furthermore, our handling of metrics introduces other constraints. To respect authorial intent, we did not average multiple metrics from a single study, which may underrepresent the full scope of some evaluations. Additionally, normalizing diverse indicators, such as accuracy scores and Likert scales, onto a unified 0 to 1 scale oversimplifies their intrinsic meanings. The review is also susceptible to search and publication bias because it was limited to English-language literature and did not include unpublished research. Finally, given the rapid advancement of the LLM field, our findings should be interpreted as a snapshot in time, which may not reflect the latest technological or methodological developments.

### Conclusions

In conclusion, the current evidence base is insufficiently comprehensive to support claims of deployment readiness for LLMs in high-stakes clinical environments. The rapid growth in accuracy-centered publications can create a misleading perception of technological readiness. To move forward, the research community must pivot from simplistic performance benchmarks to comprehensive, multidimensional evaluations grounded in established frameworks for trustworthy AI. Future studies must prioritize the development and application of robust metrics for safety, fairness, privacy, and reliability to help ensure that medical LLMs fulfill their promise without compromising patient well-being.

## Supplementary material

10.2196/97240 Multimedia Appendix 1 Correspondence between the AI Guidelines for Business and health-specific AI frameworks (World Health Organization, Consolidated Standards of Reporting Trials–AI, Standard Protocol Items: Recommendations for Interventional Trials–AI, Developmental and Exploratory Clinical Investigation of Decision Support Systems Driven by AI, Fairness, Universality, Traceability, Usability, Robustness, and Explainability for AI, Transparent Reporting of a Multivariable Prediction Model for Individual Prognosis or Diagnosis+AI).

10.2196/97240 Multimedia Appendix 2 Complete database-specific search strategies for the PubMed, Scopus, Web of Science, arXiv, and IEEE Xplore databases, including the search date (January 15, 2025) and records retrieved per database.

10.2196/97240 Multimedia Appendix 3 Metric-family performance summaries and model-selection sensitivity analyses (best, mean, median, and primary models), including cluster bootstrap CIs and the preprint-exclusion sensitivity analysis.

10.2196/97240 Multimedia Appendix 4 Prompt used for structured large language model–assisted data extraction, including the model (GPT-5 mini), access date (September 2025), and default decoding temperature.

10.2196/97240 Multimedia Appendix 5 Category-keyword correspondence and coding manual for mapping extracted terms to trustworthy AI domains, including coding rules and borderline-case examples.

10.2196/97240 Multimedia Appendix 6 Objective Reporting Transparency Index used as a fully automated surrogate for risk-of-bias assessment, including per-study index and higher-transparency sensitivity analysis.

10.2196/97240 Checklist 1 PRISMA 2020 checklist.

## Abbreviations

AUC: area under the curve
CLAIM: Checklist for Artificial Intelligence in Medical Imaging
CONSORT: Consolidated Standards of Reporting Trials
FDA: Food and Drug Administration
FUTURE: Fairness, Universality, Traceability, Usability, Robustness, and Explainability
LLM: large language model
ML: machine learning
OSF: Open Science Framework
PRISMA: Preferred Reporting Items for Systematic Reviews and Meta-Analyses
PROBAST: Prediction Model Risk of Bias Assessment Tool
RTI: Reporting Transparency Index
SMOG: Simple Measure of Gobbledygook
SPIRIT: Standard Protocol Items: Recommendations for Interventional Trials
STARD: Standards for Reporting Diagnostic Accuracy Studies
TRIPOD: Transparent Reporting of a Multivariable Prediction Model for Individual Prognosis or Diagnosis
WHO: World Health Organization

## References

[1] Lee P, Bubeck S, Petro J (2023). Benefits, limits, and risks of GPT-4 as an AI chatbot for medicine. N Engl J Med.

[2] Singhal K, Azizi S, Tu T (2023). Large language models encode clinical knowledge. Nature.

[3] Mesko B (2017). The role of artificial intelligence in precision medicine. Expert Rev Precis Med Drug Dev.

[4] Wang B, Lai J, Cao H (2024). Enhancing the interoperability and transparency of real-world data extraction in clinical research: evaluating the feasibility and impact of a ChatGLM implementation in Chinese hospital settings. Eur Heart J Digit Health.

[5] Thirunavukarasu AJ, Ting DS, Elangovan K, Gutierrez L, Tan TF, Ting DS (2023). Large language models in medicine. Nat Med.

[6] Shoja MM, Van de Ridder JM, Rajput V (2023). The emerging role of generative artificial intelligence in medical education, research, and practice. Cureus.

[7] Sallam M (2023). ChatGPT utility in healthcare education, research, and practice: systematic review on the promising perspectives and valid concerns. Healthcare (Basel).

[8] Davenport T, Kalakota R (2019). The potential for artificial intelligence in healthcare. Future Healthc J.

[9] Reddy S, Allan S, Coghlan S, Cooper P (2020). A governance model for the application of AI in health care. J Am Med Inform Assoc.

[10] Ghassemi M, Oakden-Rayner L, Beam AL (2021). The false hope of current approaches to explainable artificial intelligence in health care. Lancet Digit Health.

[11] Kung TH, Cheatham M, Medenilla A (2023). Performance of ChatGPT on USMLE: potential for AI-assisted medical education using large language models. PLOS Digit Health.

[12] Gilson A, Safranek CW, Huang T (2023). How does ChatGPT perform on the United States Medical Licensing Examination (USMLE)? The implications of large language models for medical education and knowledge assessment. JMIR Med Educ.

[13] He J, Baxter SL, Xu J, Xu J, Zhou X, Zhang K (2019). The practical implementation of artificial intelligence technologies in medicine. Nat Med.

[14] Zhang J, Zhang ZM (2023). Ethics and governance of trustworthy medical artificial intelligence. BMC Med Inform Decis Mak.

[15] Waldock WJ, Zhang J, Guni A, Nabeel A, Darzi A, Ashrafian H (2024). The accuracy and capability of artificial intelligence solutions in health care examinations and certificates: systematic review and meta-analysis. J Med Internet Res.

[16] Shool S, Adimi S, Saboori Amleshi R, Bitaraf E, Golpira R, Tara M (2025). A systematic review of large language model (LLM) evaluations in clinical medicine. BMC Med Inform Decis Mak.

[17] Asgari E, Montaña-Brown N, Dubois M (2025). A framework to assess clinical safety and hallucination rates of LLMs for medical text summarisation. NPJ Digit Med.

[18] Moëll B, Sand Aronsson F (2025). Harm reduction strategies for thoughtful use of large language models in the medical domain: perspectives for patients and clinicians. J Med Internet Res.

[19] Campellone TR, Flom M, Montgomery RM (2025). Safety and user experience of a generative artificial intelligence digital mental health intervention: exploratory randomized controlled trial. J Med Internet Res.

[20] Mahmoudi H, Chang D, Lee H, Ghaffarzadegan N, Jalali MS (2025). Critical assessment of large language models’ (ChatGPT) performance in data extraction for systematic reviews: exploratory study. JMIR AI.

[21] Chelli M, Descamps J, Lavoué V (2024). Hallucination rates and reference accuracy of ChatGPT and Bard for systematic reviews: comparative analysis. J Med Internet Res.

[22] Liu X, Cruz Rivera S, Moher D, Calvert MJ, Denniston AK, SPIRIT-AI and CONSORT-AI Working Group (2020). Reporting guidelines for clinical trial reports for interventions involving artificial intelligence: the CONSORT-AI extension. Nat Med.

[23] Cruz Rivera S, Liu X, Chan AW, Denniston AK, Calvert MJ, SPIRIT-AI and CONSORT-AI Working Group (2020). Guidelines for clinical trial protocols for interventions involving artificial intelligence: the SPIRIT-AI extension. Lancet Digit Health.

[24] Lekadir K, Frangi AF, Porras AR (2025). FUTURE-AI: international consensus guideline for trustworthy and deployable artificial intelligence in healthcare. BMJ.

[25] Corrêa NK, Galvão C, Santos JW (2023). Worldwide AI ethics: a review of 200 guidelines and recommendations for AI governance. Patterns (N Y).

[26] (2024). AI guidelines for business ver 1.0 compiled. Ministry of Internal Affairs and Communications Japan; Ministry of Economy, Trade and Industry.

[27] Proposal for a regulation of the European Parliament and of the Council laying down harmonised rules on artificial intelligence (Artificial Intelligence Act) and amending certain Union legislative acts. EU AI Act.

[28] Artificial intelligence in software as a medical device. U.S. Food & Drug Administration.

[29] (2021). Ethics and governance of artificial intelligence for health: WHO guidance. World Health Organization.

