# Supplementary material for: Safety-Oriented Evaluation of Large Language Models in Health Care: Guideline-Informed Systematic Review
Source: Online J Public Health Inform. 2026 Aug 20;18:e97240. doi: 10.2196/97240 (PMC13492481; doi:10.2196/97240)
Supplement: Supplementary file 1 [file ojphi-v18-e97240-s001.docx]

**Multimedia Appendix 1. Correspondence between the AI Guidelines for Business and health-specific AI frameworks**

Each trustworthy-AI domain used in this review (left) is mapped to corresponding principles or reporting items in health-specific AI guidance, supporting the use of the AI Guidelines for Business as a complementary cross-sectoral organizing scaffold.

| **Domain (this review)** | **WHO guidance** | **CONSORT-AI / SPIRIT-AI** | **FUTURE-AI / DECIDE-AI** | **TRIPOD+AI** |
| --- | --- | --- | --- | --- |
| Accuracy | Effective performance | Outcomes; AI performance | Robustness; Usability | Model performance; calibration |
| Fairness & inclusiveness | Equity; non-discrimination | Subgroup/handling of inputs | Fairness | Fairness; subgroup analysis |
| Data quality | Data governance | Input data handling/quality | Universality; data quality | Data sources; quality |
| Prevention of misinformation | Safety; reliability | Error/poor-quality output handling | Traceability | Outcome definition |
| Control of harmful information | Safety; do no harm | Adverse events/errors | Robustness; safety | — |
| Management of high-risk uses | Risk management; oversight | Intended use; settings | Usability; lifecycle | Intended use |
| Robustness | Reliability | Performance under variation | Robustness | Validation; stability |
| Explainability | Transparency; explicability | Explanation of AI outputs | Explainability | Interpretability |
| Verifiability | Accountability; auditability | Reproducibility; access | Traceability | Reproducibility; reporting |
| Privacy protection | Privacy; data protection | Data handling/consent | Privacy by design | Data privacy |
| Security assurance | Security; safety | Security of system | Robustness; security | — |
