# Supplementary material for: Safety-Oriented Evaluation of Large Language Models in Health Care: Guideline-Informed Systematic Review
Source: Online J Public Health Inform. 2026 Aug 20;18:e97240. doi: 10.2196/97240 (PMC13492481; doi:10.2196/97240)
Supplement: Supplementary file 2 [file ojphi-v18-e97240-s002.docx]

**Table S1. The complete search syntax for four databases**

| Database name | Search syntax | Number of results |
| --- | --- | --- |
| PubMed | ("Large language model"[ti] OR "Large language models"[ti] OR (large[ti] AND "language model"[ti]) OR (large[ti] AND "language models"[ti]) OR ("large language"[ti] AND model[ti]) OR ("large language"[ti] AND models[ti]) OR "language model"[ti] OR "language models"[ti] OR LLM[ti] OR LLMs[ti] OR "GPT models"[ti] OR "GPT model"[ti] OR Gpt[ti] OR gpts[ti] OR Chatgpt[ti] OR "generative pre-trained transformer"[ti] OR "bidirectional encoder representations from transformers"[ti] OR BERT[ti] OR "transformer-based model"[ti] OR (transformer[ti] AND model[ti]) OR (transformers[ti] AND model[ti]) OR (transformer[ti] AND models[ti]) OR (transformers[ti] AND models[ti])) AND (Evaluation[ti] OR Evaluat*[ti] OR Assessment[ti] OR Assess*[ti] OR Validation[ti] OR Validat*[ti] OR Benchmarking[ti] OR Benchmark*[ti]) | 1201 |
| Scopus | (TITLE("Large language model") OR TITLE("Large language models") OR (TITLE(large) AND TITLE("language model")) OR (TITLE(large) AND TITLE("language models")) OR (TITLE("large language") AND TITLE(model)) OR (TITLE("large language") AND TITLE(models)) OR TITLE("language model") OR TITLE("language models") OR TITLE(LLM) OR TITLE(LLMs) OR TITLE("GPT models") OR TITLE("GPT model") OR TITLE(Gpt) OR TITLE(gpts) OR TITLE(Chatgpt) OR TITLE("generative pre-trained transformer") OR TITLE("bidirectional encoder representations from transformers") OR TITLE(BERT) OR TITLE("transformer-based model") OR (TITLE(transformer) AND TITLE(model)) OR (TITLE(transformers) AND TITLE(model)) OR (TITLE(transformer) AND TITLE(models)) OR (TITLE(transformers) AND TITLE(models))) AND (TITLE(Evaluation) OR TITLE(Evaluat*) OR TITLE(Assessment) OR TITLE(Assess*) OR TITLE(Validation) OR TITLE(Validat*) OR TITLE(Benchmarking) OR TITLE(Benchmark*)) | 3273 |
| WoS | (TI=("Large language model") OR TI=("Large language models") OR (TI=(large) AND TI=("language model")) OR (TI=(large) AND TI=("language models")) OR (TI=("large language") AND TI=(model)) OR (TI=("large language") AND TI=(models)) OR TI=("language model") OR TI=("language models") OR TI=(LLM) OR TI=(LLMs) OR TI=("GPT models") OR TI=("GPT model") OR TI=(Gpt) OR TI=(gpts) OR TI=(Chatgpt) OR TI=("generative pre-trained transformer") OR TI=("bidirectional encoder representations from transformers") OR TI=(BERT) OR TI=("transformer-based model") OR (TI=(transformer) AND TI=(model)) OR (TI=(transformers) AND TI=(model)) OR (TI=(transformer) AND TI=(models)) OR (TI=(transformers) AND TI=(models))) AND (TI=(Evaluation) OR TI=(Evaluat*) OR TI=(Assessment) OR TI=(Assess*) OR TI=(Validation) OR TI=(Validat*) OR TI=(Benchmarking) OR TI=(Benchmark*)) | 1989 |
| arXiv | (ti:“Large language model” OR ti:“Large language models” OR (ti:large AND ti:“language model”) OR (ti:large AND ti:“language models”) OR (ti:“large language” AND ti:model) OR (ti:“large language” AND ti:models) OR ti:“language model” OR ti:“language models” OR ti:LLM OR ti:LLMs OR ti:“GPT models” OR ti:“GPT model” OR ti:Gpt OR ti:gpts OR ti:Chatgpt OR ti:“generative pre-trained transformer” OR ti:“bidirectional encoder representations from transformers” OR ti:BERT OR ti:“transformer-based model” OR (ti:transformer AND ti:model) OR (ti:transformers AND ti:model) OR (ti:transformer AND ti:models) OR (ti:transformers AND ti:models)) AND (ti:Evaluation OR ti:Evaluat* OR ti:Assessment OR ti:Assess* OR ti:Validation OR ti:Validat* OR ti:Benchmarking OR ti:Benchmark*) | 18128 |
| IEEE Xplore | (("Document Title":"Large language model" OR title:"Large language models" OR ("Document Title":"large" AND "Document Title":"language model") OR ("Document Title":"large" AND "Document Title":"language models") OR ("Document Title":"large language" AND "Document Title":"model") OR ("Document Title":"large language" AND "Document Title":"models") OR title:"language model" OR title:"language models" OR title:"LLM" OR title:"LLMs" OR title:"GPT models" OR title:"GPT model" OR title:"Gpt" OR title:"gpts" OR title:"Chatgpt" OR title:"generative pre-trained transformer" OR title:"bidirectional encoder representations from transformers" OR title:"BERT" OR title:"transformer-based model" OR ("Document Title":"transformer" AND "Document Title":"model") OR ("Document Title":"transformers" AND "Document Title":"model") OR ("Document Title":"transformer" AND "Document Title":"models") OR ("Document Title":"transformers" AND "Document Title":"models")) AND ("Document Title":"Evaluation" OR title:"Evaluat*" OR title:"Assessment" OR title:"Assess*" OR title:"Validation" OR title:"Validat*" OR title:"Benchmarking" OR title:"Benchmark*") ) | 565 |
