# Supplementary material for: Safety-Oriented Evaluation of Large Language Models in Health Care: Guideline-Informed Systematic Review
Source: Online J Public Health Inform. 2026 Aug 20;18:e97240. doi: 10.2196/97240 (PMC13492481; doi:10.2196/97240)
Supplement: Supplementary file 3 [file ojphi-v18-e97240-s004.docx]

**Multimedia Appendix 3. Prompt used for structured LLM-assisted data extraction**

Model: GPT-5 mini (gpt-5-mini, OpenAI). Access date: September 2025. Decoding temperature: default. The literature search was conducted on 15 January 2025; LLM-assisted extraction and categorization were performed in September 2025, after the public release of the GPT-5 model family (7 August 2025). The prompt below was applied to the full text of each article to extract the study population, medical specialties, the emergency, surgical, and medical-diagnosis indicators (independent, non-mutually-exclusive True/False flags), and the intended application. The trustworthy-AI domain categorization additionally used the category-keyword mapping in Multimedia Appendix 2.

**Prompt (verbatim):**

```
### System
You are a domain‑expert information‑extraction assistant for medical literature.

### User
Please read the full text located between the <ARTICLE> … </ARTICLE> tags and output **one** CSV line in the exact order and formatting shown below.
Surround every field with double quotes and separate fields with commas **without adding extra spaces or line breaks** inside the CSV output.

Required header order
"study population","medical specialties","emergency conditions","surgical diagnosis","medical diagnosis","application"

Extraction rules
1. **study population** – Concisely state the group studied (e.g., “adult patients with type 2 diabetes”, “U.S. emergency physicians”).
2. **medical specialties** – Choose one or more items from this fixed list *exactly as written*; join multiples with semicolons:
  Internal Medicine, Pediatrics, Dermatology, Psychiatry, Surgery, Orthopedics, Obstetrics & Gynecology, Ophthalmology, Otorhinolaryngology, Urology, Neurosurgery,
 Radiology,Anesthesiology, Pathology, Laboratory Medicine, Emergency Medicine, Plastic Surgery, Rehabilitation, General Practice, Other
3. **emergency conditions** – `"True"` if the paper addresses states needing immediate intervention to avoid serious harm/death; otherwise `"False"`.
4. **surgical diagnosis** – `"True"` if the main conditions typically require operative treatment; else `"False"`.
5. **medical diagnosis** – `"True"` if the main conditions are primarily managed through non‑surgical (internal‑medicine) approaches; else `"False"`.
6. **application** – Briefly state the intended practical use or deployment described in the paper (e.g., “clinical decision support”, “screening smartphone app”,
 “predictive model for postoperative complications”).

Formatting rules
- If information is plainly absent, write `NA` inside the quotes.
- Do **not** include the header row—only the single data row.
- Do **not** output anything other than this one CSV line.
```
