# Supplementary material for: Safety-Oriented Evaluation of Large Language Models in Health Care: Guideline-Informed Systematic Review
Source: Online J Public Health Inform. 2026 Aug 20;18:e97240. doi: 10.2196/97240 (PMC13492481; doi:10.2196/97240)
Supplement: Supplementary file 4 [file ojphi-v18-e97240-s005.docx]

**Multimedia Appendix 2. Category–keyword correspondence and coding manual**

This coding manual specifies how extracted terms and reported evaluation metrics were mapped to the trustworthy-AI evaluation domains. The category-keyword correspondence below was applied by the LLM-assisted categorization (GPT-5) together with rule-based keyword matching; the resulting assignments were validated by fully automated procedures (Multimedia Appendix 7).

## Category–keyword correspondence

| **Domain (this review)** | **Japanese category** | **Mapped keywords / terms (case-insensitive, stem-matched)** |
| --- | --- | --- |
| (Overall) Safety | 全体 | Safety |
| Control of harmful information | 有害情報の出力制御 | Harm |
| Prevention of misinformation | 誤情報の出力防止・誘導の防止 | Misuse; Misinformation; Usefulness; Relevance; Reliability |
| Fairness & inclusiveness | 公平性と包摂性 | Bias; Fair; Acceptability; Concise; Comprehensive |
| Management of high-risk uses | ハイリスク利用・目的外への対処 | Risk |
| Privacy protection | プライバシー保護 | Privacy |
| Security assurance | セキュリティ確保 | Security |
| Explainability | 説明可能性 | Readability; Transparent; Transparency; Visibility; Understand; Clarity |
| Robustness | ロバスト性 | Robust; Consistency; Coherence; Redundancy |
| Data quality | データ品質 | Quality; Complete |
| Verifiability | 検証可能性 | Verification; Visibility; Actionability; Reference |

Accuracy (correctness against a reference; e.g., accuracy, AUC, F1, % correct) was treated as a distinct performance domain and is not listed above.

## Coding rules

1. Matching was case-insensitive and stem-based (e.g., “fair” matches “fairness”, “unfair”). 2. Multi-label assignment was allowed: a study could be assigned to several domains. 3. When a term appeared under more than one category (e.g., “Visibility” under both Explainability and Verifiability), the assignment was made according to the primary construct the metric measured in that study, and ambiguous cases were adjudicated. 4. Readability indices (e.g., Flesch-Kincaid, SMOG) were mapped to Explainability when used to assess the interpretability/understandability of outputs, and to Data quality when used as an output-quality descriptor. 5. Terms not matching any category were left unassigned (counted as “none”).

## Borderline examples

| **Situation** | **Resolution** |
| --- | --- |
| A study reports a bias/subgroup metric and a fairness Likert rating | Assigned to Fairness & inclusiveness (both terms map to the same domain). |
| “Visibility” used to describe source citation | Assigned to Verifiability (source traceability), not Explainability. |
| Readability index used to judge patient-facing clarity | Assigned to Explainability (understandability of outputs). |
| “Reliability” used as test-retest stability | Assigned to Robustness when describing stability; to Prevention of misinformation when describing factual reliability (adjudicated by context). |
| A consistency/coherence metric across prompts | Assigned to Robustness. |
