# Supplementary material for: Safety-Oriented Evaluation of Large Language Models in Health Care: Guideline-Informed Systematic Review
Source: Online J Public Health Inform. 2026 Aug 20;18:e97240. doi: 10.2196/97240 (PMC13492481; doi:10.2196/97240)
Supplement: Supplementary file 5 [file ojphi-v18-e97240-s007.docx]

# **PRISMA 2020 Checklist**

*Safety-Oriented Evaluation of Large Language Models in Health Care: Guideline-Informed Systematic Review*

Online Journal of Public Health Informatics, manuscript 97240. Page numbers refer to the copyedited proof.

| **Section and topic** | **Item #** | **Checklist item** | **Location where item is reported** |
| --- | --- | --- | --- |
| **TITLE** | | | |
| Title | 1 | Identify the report as a systematic review. | Title (p 1) |
| **ABSTRACT** | | | |
| Abstract | 2 | See the PRISMA 2020 for Abstracts checklist. | Abstract (p 1) |
| **INTRODUCTION** | | | |
| Rationale | 3 | Describe the rationale for the review in the context of existing knowledge. | Introduction (p 2) |
| Objectives | 4 | Provide an explicit statement of the objective(s) or question(s) the review addresses. | Abstract, Objective (p 1); Introduction, final two paragraphs (p 2) |
| **METHODS** | | | |
| Eligibility criteria | 5 | Specify the inclusion and exclusion criteria for the review and how studies were grouped for the syntheses. | Methods, Systematic Review Data Extraction and Management Protocol (p 3); Table 1 (p 5) |
| Information sources | 6 | Specify all databases, registers, websites, organisations, reference lists and other sources searched or consulted to identify studies. Specify the date when each source was last searched or consulted. | Methods (p 3): PubMed, Scopus, Web of Science, arXiv and IEEE Xplore, all searched on January 15, 2025; Multimedia Appendix 2 |
| Search strategy | 7 | Present the full search strategies for all databases, registers and websites, including any filters and limits used. | Methods (p 3); full search schema in Multimedia Appendix 2 |
| Selection process | 8 | Specify the methods used to decide whether a study met the inclusion criteria of the review, including how many reviewers screened each record and each report retrieved, whether they worked independently, and if applicable, details of automation tools used in the process. | Methods (p 3): two-stage screening (title and abstract, then full text) by 3 reviewers working together, with records retained only by unanimous consensus. Limitations (p 9): screening was not independent or duplicated |
| Data collection process | 9 | Specify the methods used to collect data from reports, including how many reviewers collected data from each report, whether they worked independently, any processes for obtaining or confirming data from investigators, and if applicable, details of automation tools used in the process. | Methods, Data Classification and Categorization (p 4): LLM-assisted extraction (GPT-5 mini) with an automated internal consistency check; Table 2 (p 8); full prompt in Multimedia Appendix 4 |
| Data items | 10a | List and define all outcomes for which data were sought. Specify whether all results that were compatible with each outcome domain in each study were sought, and if not, the methods used to decide which results to collect. | Methods, Approach to Score Calculation and Normalization (p 4); Table 2 (p 8); Multimedia Appendix 3 |
|  | 10b | List and define all other variables for which data were sought. Describe any assumptions made about any missing or unclear information. | Methods, Data Classification and Categorization (p 4); Table 2, structured data extraction schema (p 8) |
| Study risk of bias assessment | 11 | Specify the methods used to assess risk of bias in the included studies, including details of the tool(s) used, how many reviewers assessed each study and whether they worked independently, and if applicable, details of automation tools used in the process. | Methods, Data Classification and Categorization (p 4): conventional risk-of-bias tools were not applicable at this scale; an objective Reporting Transparency Index (RTI, score range 0-5) was derived instead. Multimedia Appendix 6 |
| Effect measures | 12 | Specify for each outcome the effect measure(s) (e.g. risk ratio, mean difference) used in the synthesis or presentation of results. | Methods, Approach to Score Calculation and Normalization (p 4): metrics normalised to a 0-1 scale; study-aggregated mean normalised score with 95% CI |
| Synthesis methods | 13a | Describe the processes used to decide which studies were eligible for each synthesis. | Methods (p 4): summarised within metric families; fundamentally different families were not pooled; categories with <5 valid items excluded from interval estimation |
|  | 13b | Describe any methods required to prepare the data for presentation or synthesis, such as handling of missing summary statistics, or data conversions. | Methods, Approach to Score Calculation and Normalization (p 4): normalisation of accuracy, AUC and F1-score; rescaling of Likert-type scales; handling of readability indices |
|  | 13c | Describe any methods used to tabulate or visually display results of individual studies and syntheses. | Results: Figures 2-5 (p 5-8); Tables 1-2 (p 5, 8); Multimedia Appendix 3 |
|  | 13d | Describe any methods used to synthesize results and provide a rationale for the choice(s). If meta-analysis was performed, describe the model(s), method(s) to identify the presence and extent of statistical heterogeneity, and software package(s) used. | Methods (p 4): the analysis was descriptive and not a formal meta-analysis; one value per study per domain to avoid pseudoreplication; 95% CIs estimated by cluster bootstrap resampling of studies |
|  | 13e | Describe any methods used to explore possible causes of heterogeneity among study results (e.g. subgroup analysis, meta-regression). | Methods (p 4) and Results, Evaluations Within Each Branch of Medicine (p 6-7): metric families reported separately; subgroup analyses by branch of medicine (Figure 4) |
|  | 13f | Describe any methods used to assess robustness of the synthesized results. | Methods (p 4) and Results (p 5): best, median and primary-model sensitivity analyses; RTI-restricted analysis; preprint-exclusion analysis. Multimedia Appendices 3 and 6 |
| Reporting bias assessment | 14 | Describe any methods used to assess risk of bias due to missing results in a synthesis (arising from reporting biases). | Formal reporting-bias assessment (e.g. funnel plots) was not applicable because the synthesis was descriptive. Sources of potential reporting bias are addressed in Limitations (p 9-10) |
| Certainty assessment | 15 | Describe any methods used to assess certainty (or confidence) in the body of evidence for an outcome. | GRADE was not applied. Precision was conveyed by 95% CIs from cluster bootstrap, and reporting completeness by the RTI (Methods, p 4) |
| **RESULTS** | | | |
| Study selection | 16a | Describe the results of the search and selection process, from the number of records identified in the search to the number of studies included in the review, ideally using a flow diagram. | Results reported in Methods narrative (p 3) and Figure 1, PRISMA 2020 flow diagram (p 3) |
|  | 16b | Cite studies that might appear to meet the inclusion criteria, but which were excluded, and explain why they were excluded. | Reasons for exclusion at each stage are given in Figure 1 (p 3) and in the accompanying narrative (p 3) |
| Study characteristics | 17 | Cite each included study and present its characteristics. | Characteristics are summarised across domains and specialties in Figures 2 and 5 and Tables 1-2; per-study characteristics are provided in Multimedia Appendix 3 |
| Risk of bias in studies | 18 | Present assessments of risk of bias for each included study. | RTI scores per study are reported in Multimedia Appendix 6 |
| Results of individual studies | 19 | For all outcomes, present, for each study, summary statistics for each group (where appropriate) and an effect estimate and its precision, ideally using structured tables or plots. | Study-level normalised values are provided in Multimedia Appendix 3 |
| Results of syntheses | 20a | For each synthesis, briefly summarise the characteristics and risk of bias among contributing studies. | Results, Distribution of Categories (p 4-5) and Category-Wise Numerical Evaluation (p 5-6); Figure 2 (p 5) |
|  | 20b | Present results of all statistical syntheses conducted. If meta-analysis was done, present for each the summary estimate and its precision (e.g. confidence/credible interval) and measures of statistical heterogeneity. | Results, Category-Wise Numerical Evaluation (p 5-6); Figure 3 (p 6): study-aggregated means with 95% CIs for each domain |
|  | 20c | Present results of all investigations of possible causes of heterogeneity among study results. | Results, Evaluations Within Each Branch of Medicine (p 6-7); Figure 4 (p 7); metric-family results in Multimedia Appendix 3 |
|  | 20d | Present results of all sensitivity analyses conducted to assess the robustness of the synthesized results. | Results (p 5): best-model selection changed domain means by 0.01-0.05; RTI-restricted analysis changed estimates by at most 0.004; preprint exclusion by at most 0.018. Multimedia Appendices 3 and 6 |
| Reporting biases | 21 | Present assessments of risk of bias due to missing results (arising from reporting biases) for each synthesis assessed. | Discussion, Limitations (p 9-10): restriction to English-language and published literature is identified as a source of search and publication bias |
| Certainty of evidence | 22 | Present assessments of certainty (or confidence) in the body of evidence for each outcome assessed. | Results, Category-Wise Numerical Evaluation (p 5-6): wide CIs for domains with few valid items are interpreted as reduced certainty; Discussion, Limitations (p 9-10) |
| **DISCUSSION** | | | |
| Discussion | 23a | Provide a general interpretation of the results in the context of other evidence. | Discussion, Principal Results (p 9) |
|  | 23b | Discuss any limitations of the evidence included in the review. | Discussion, Limitations (p 9-10) |
|  | 23c | Discuss any limitations of the review processes used. | Discussion, Limitations (p 9-10): LLM-assisted categorisation validated without an independent human gold standard; retrospective registration; screening not independent or duplicated |
|  | 23d | Discuss implications of the results for practice, policy, and future research. | Discussion, Principal Results and Conclusions (p 9) |
| **OTHER INFORMATION** | | | |
| Registration and protocol | 24a | Provide registration information for the review, including register name and registration number, or state that the review was not registered. | Methods (p 3): retrospectively registered on the Open Science Framework, DOI 10.17605/OSF.IO/P4KSB, registered on June 22, 2026 and labelled retrospective |
|  | 24b | Indicate where the review protocol can be accessed, or state that a protocol was not prepared. | The registration record is available at DOI 10.17605/OSF.IO/P4KSB (Methods, p 3). A prospective protocol was not prepared |
|  | 24c | Describe and explain any amendments to information provided at registration or in the protocol. | Not applicable. Registration was retrospective and no amendments were made |
| Support | 25 | Describe sources of financial or non-financial support for the review, and the role of the funders or sponsors in the review. | Funding: the authors declared no financial support was received for this work |
| Competing interests | 26 | Declare any competing interests of review authors. | Conflicts of Interest: none declared |
| Availability of data, code and other materials | 27 | Report which of the following are publicly available and where they can be found: template data collection forms; data extracted from included studies; data used for all analyses; analytic code; any other materials used in the review. | Multimedia Appendix 2 (search schema), Appendix 3 (metric-family summaries and sensitivity analyses), Appendix 4 (extraction schema and full LLM prompt), Appendix 5 (coding manual), Appendix 6 (RTI data) |

*From: Page MJ, McKenzie JE, Bossuyt PM, Boutron I, Hoffmann TC, Mulrow CD, et al. The PRISMA 2020 statement: an updated guideline for reporting systematic reviews. BMJ. 2021;372:n71. doi: 10.1136/bmj.n71*
